# What to Eat

**Published:** 1888-12

**Authors:** S. H. Preston


### WHAT TO EAT.

BY S. H. PRESTON.

[Reply to Remarks on Right Living in November Number of JOURNAL OF HEALTH, page 243.]

As applied to diet, a grain of common sense discounts a great deal of chemistry. Experience proves to be a better guide in eating than any physician. There is something about the stomach that upsets the analysis of science and refutes all the rules of the alleged food reformer.

For instance : Here is the Hon. Hygiene Abstemious prostrated with dyspepsia after practicing the most correct and physiological course of living. Doctors do him no good. Indeed, the more he doctors the worse he gets. The medical counsellors say he must eat nothing but milk. That is the only approved diet for dyspepsia. Well, strange to say, the patient loathes milk now, albeit he always liked it before. Although he heretofore abhorred pork and pickles, and such pernicious things, he craves them now. The physicians say this is due to some strange perversity of his dyspeptic condition. He never wanted anything in his life so much as a plate of pork and beans. His doctors declare that nothing would kill him so surely as that. But he decides that he had better die than be denied pork and beans. So he gets them, and eats enough to bluff a Bostonian, and begins to feel better. Then he makes a raid on the medicine vials and bottles till they are all abolished in the ash barrel. And this gives him an appetite for more pork and beans, and the next day he adds corned beef and cabbage, sauer kraut and pickles, and he washes the whole down with an abundance of hard cider and old ale, and thus goes on till he becomes robust and fat and fitted for the prize ring.

Now, neighbor Goodcheer, a notorious old glutton and reckless liver, is attacked with the same complaint. He tries the same pork and beans remedy, and dies a dreadful death. What cured the temperate valetudinarian killed the tough old epicure. Milk might have killed the former and cured the latter.

The fact is, there can be no prescribed regimen that will produce the same result upon two persons. What will work well in one individual instance will prove injurious in another. People differ as radically in gastronomic idiosyncracies as in temperament and features.

A universal dietary is a delusion. There is no such thing as sanitary science. So-called medical science is simply a system of experiment. Experience alone can determine the proper diet for each person. One man may derive enough nourishment from brown bread, bananas and peanuts, while another requires roast beef, pork chops and blue points. Milk for one, meat for another, according to climate, calling and condition. No need to classify foods into carbonaceous, nitrogenous, etc., according to chemical constituents. What each wants, whether lord or laborer, is that which agrees best with his appetite and digestion.

Suppose a Polish prisoner did live a long time on rye bread. That men have lived and thrived to as great an age on tobacco and alcohol does not prove that these things are conducive to health and longevity. Admit that an anthropoid ape acquires strength and vigor from forest fruits and nuts, and that a Chinaman who cannot get rats flourishes on rice ; this does not prove that fruit and nuts are better than fresh meat for the carnivorous lion, or that civilized man should change his choice and elaborate bill of fare for that of the forest ape, because of man's structural resemblance to this animal, as argued by a vegetarian advocate.

But man has changed his dress and all the conditions of life, as well as his diet, since he emerged from the primeval wilds of his apish ancestors. In his advance from chimpanzeeism to civilization he may have lost much in brawn, but he has gained greatly in brain. In forsaking that "large percentage of free oil found in nuts," which, according to the vegetarian advocate aforesaid, is so indispensable to an able-bodied ape, man may have damaged his constitution ; but perhaps the many amenities of modern life may partly compensate for this. The Digger Indian who still subsists on forest roots is undoubtedly better adapted to deal with rugged nature in a rough-handed way than the dainty diner at Delmonico's. Grant that the former will outlive the latter and preserve a sounder stomach, provided he saves his scalp ; is such a life as his worth prolonging ? Living to dig roots, and eating roots to live. As well lie rotting in the ground to fertilize roots for other animals to eat.

Every creature is characterized by what it feeds upon. The stupid sheep and subtle fox, the silly ass and lordly lion, illustrate the different influences of herbivorous and carnivorous foods in animal life. Who would not rather live ten years as a big-brained, bouyant-blooded animal, than fifty years as an inert, earth-rooted vegetable?

But it is far from a fact that fig-fed folks of the East, or the black bread eaters of Europe are healthier or more muscular than other men. The human race has no more splendid specimens of physical development, jocund health and robust manhood, than the cowboys of our Western plains and the rough rangers of the Southern pampas. In the saddle day after day, from dawn to dark, their endurance is surprising. They live upon meat—seldom see any other food than fresh meat. There breathe no more hardy or brawny men than the beef-eating soldiers of Britain. How long would the rice-rationed troops of China stand before them in a hand to hand struggle? Would Wellington have won Waterloo with men fed on peanuts and stewed prunes?

A few years ago an experiment was made with several English regiments. They were put upon a vegetable diet, the principal article being the potato. The effect was a gain in flesh, but a loss in vigor. The men became torpid and pot-bellied. It was found to be unsuitable food for soldiers.

Now, facts are sternly stubborn things, and our observation confirms the fact that a strictly cereal, fruit and nut diet, is detrimental to any class of people. The vegetarian leads a vegetable kind of life. He can be picked out from a crowd. He is skinny, tallow-faced and flabby-fleshed. There is no "speculation in his eyes," he seems half asleep, and he presents, generally, somewhat of a peanutty appearance. Talk with him and you discover that his brain is in a cloud. His memory is a mist. It requires great mental effort for him to recall his own name. This impairment of memory marks every vegetarian we meet. Here is a fact for phrenological investigation. Whole wheat bread and fruit and "free oil-nuts" may be excellent for the bowels, but they are mighty bad for the memory.

Where do we find models of mental and muscular vigor? Among vegetarians and folks who are fretting the flesh from their bones about their health? In hygienic hotels and sanitariums? Not much. See the cadaverous, consumptive looking set. Any baseball champions, bridge jumpers, or pugilists among them? Not much. Where do we find the paragons of physical perfection? I will tell you truly. About butcher's shops and lager beer saloons and in bountifully served restaurants, bolting down porterhouse steaks. Verily, facts are stubborn things.

